# An arginase‐1 SNP that protects against the development of pulmonary hypertension in bronchopulmonary dysplasia enhances NO‐mediated apoptosis in lymphocytes

**DOI:** 10.14814/phy2.13041

**Published:** 2016-11-28

**Authors:** Jennifer K. Trittmann, Yi Jin, Louis G. Chicoine, Yusen Liu, Bernadette Chen, Leif D. Nelin

**Affiliations:** ^1^ Pulmonary Hypertension GroupCenter for Perinatal ResearchThe Ohio State UniversityColumbusOhio; ^2^ Department of PediatricsThe Ohio State UniversityColumbusOhio; ^3^ Center for Gene TherapyThe Research Institute at Nationwide Children's HospitalThe Ohio State UniversityColumbusOhio

**Keywords:** Bronchopulmonary dysplasia, neonate, oxygen, preterm, urea cycle

## Abstract

Arginase and nitric oxide synthase (NOS) share a common substrate, l‐arginine, and have opposing effects on vascular remodeling. Arginase is the first step in polyamine and proline synthesis necessary for cellular proliferation, while NO produced from NOS promotes apoptosis. Previously, we identified a single nucleotide polymorphism (SNP) in the arginase‐1 (*ARG1*) gene, rs2781666 (T‐allele) that was associated with a decreased risk for developing pulmonary hypertension (PH) in a cohort of infants with bronchopulmonary dysplasia (BPD). In this study, we utilized lymphocytes from neonates (the only readily available cells from these patients expressing the two genotypes of interest) with either the rs2781666 SNP (TT) or wild type (GG) to test the hypothesis that the protection of the *ARG1* SNP against the development of PH in BPD would involve augmented NO production leading to more apoptosis. Lymphocytes were stimulated with IL‐4, IL‐13, and phorbol myristate acetate (PMA). We found that TT lymphocytes had similar levels of arginase I and arginase II expression, but there was a tendency for lower urea production (a surrogate marker of arginase activity), than in the GG lymphocytes. The TT lymphocytes also had significantly greater NO production than did GG lymphocytes despite no differences in iNOS expression between genotypes. Furthermore, the TT lymphocytes had lower numbers of viable cells, and higher levels of cleaved caspase‐3 than did GG lymphocytes. Inhibiting NOS activity using *N*_*ω*_‐Nitro‐l‐arginine methyl ester hydrochloride (l‐NAME) significantly decreased cleaved caspase‐3 levels in the TT lymphocytes. These data demonstrate that the TT genotype results in greater levels of NO production leading to more apoptosis, which is consistent with the concept that BPD patients with the TT genotype are protected against the development of PH by producing greater basal levels of endogenous NO.

## Introduction

Bronchopulmonary dysplasia (BPD) is seen primarily in extremely preterm neonates who require positive pressure ventilation and supplemental oxygen in order to survive. However, these therapies can result in disruption of normal lung development leading to BPD and BPD‐associated pulmonary hypertension (PH) (Baker et al. 2014). Nitric oxide (NO) is a powerful vasodilator that is endogenously produced from l‐arginine by NO synthase (NOS) (Wu and Morris 1998). l‐arginine can also be metabolized by arginase to produce l‐ornithine and urea. l‐ornithine is further converted to proline and polyamines, essential for the cellular proliferation that leads to pulmonary vascular remodeling in PH (Morris 2009).

Arginase has two isoforms which are encoded by two distinct genes: arginase‐1 (*ARG1)* on chromosome 6 (Sparkes et al. 1986) and arginase‐2 (*ARG2*) on chromosome 14 (Gotoh et al. 1997). The induction of arginase is involved in many disease states, including asthma (Morris et al. 2004; Morris 2012), sickle cell disease (Morris et al. 2005; Morris 2012), vascular diseases (White et al. 2006; Morris 2012), and PH (Grasemann et al. 2015; Nara et al. 2015; Steppan et al. 2016). We have previously shown that NOS and arginase compete for their common substrate, l‐arginine, such that greater expression and/or activity of one results in lower activity of the other due to limitations in l‐arginine bioavailability (Nelin et al. 2002, 2007; Chicoine et al. 2004; Stanley et al. 2006). Decreased NO production in the pulmonary circulation, as seen in some forms of PH (Xu et al. 2004), leads to vasoconstriction and greater vascular proliferation resulting in the pulmonary vascular remodeling that is a hallmark of the disease (Baker et al. 2014).

Previously, we studied 17 single nucleotide polymorphisms (SNPs) in the l‐arginine/NO pathway genes in a cohort of infants with BPD stratified by PH status. We found that one SNP, a G to T substitution at position 4195 in the *ARG1* gene (*ARG1* rs2781666 SNP), was less frequent in patients with BPD and PH than in patients with BPD alone, and for each copy of the SNP minor allele (T), the odds of developing PH decreased by 43% (Trittmann et al. 2014). We speculate that the rs2781666 SNP could potentially alter arginase I function resulting in less l‐arginine utilization by the mutated arginase I, potentially resulting in greater NO production due to greater l‐arginine bioavailability to NOS. If greater endogenous NO production was present, then this could protect against the development of PH in BPD by preventing vascular remodeling through greater NO‐mediated apoptosis (Brune et al. 1999). Therefore, the objective of this study was to determine if the presence of the *ARG1* rs2781666 SNP resulted in greater NO production in cells isolated from patients. It is very difficult to isolate cells from neonates due to size restrictions, therefore the only cells from neonatal patients that we had access to were lymphocytes isolated from umbilical cord blood at the time of delivery.

## Materials and Methods

### Human lymphocyte culture

We utilized patient cord blood specimens from our Perinatal Research Repository (PRR) to isolate and immortalize B lymphocytes, which we then studied in vitro. Lymphocytes were isolated from neonatal cord blood by centrifugation using the Ficoll‐Paque technique. White blood cells were pipetted off and then washed with a neutral buffer, resulting in a suspension of T lymphocytes, B lymphocytes, and natural killer cells. This suspension was then transfected with a modified Epstein–Barr virus, infecting only B lymphocytes. Transformed B lymphocyte cell lines were created for each neonatal cord blood specimen in RPMI‐1640 media, containing 200 mg/L l‐arginine. For all experiments, lymphocytes were stimulated with 25 ng/mL phorbol myristate acetate (PMA), 2 ng/mL interleukin‐4 (IL‐4), and 10 ng/mL IL‐13 and incubated in 21% O_2_‐5% CO_2_‐balance N_2_ for 24 h.

### Genotyping

Lymphocytes were classified as either homozygous *ARG1* rs2781666 SNP (TT) or homozygous *ARG1* rs2781666 (GG) wild type. DNA was isolated from each lymphocyte. PCR was used to determine the genotype status of the rs2781666 allele, using a Taqman SNP genotyping assay kit (Thermo Fisher Scientific, Grand Island, NY). In order to examine potential allelic differences, only lymphocytes homozygous for the TT or the GG genotypes were used in the subsequent analyses (*ARG1*: OMIM: 608313; NCBI Reference Sequence: NG_007086.2:g.4195G<T).

### Human lymphocyte RNA isolation

RNA was isolated as previously described (Nelin et al. 2007). Briefly, 1 mL of Trizol (Thermo Fisher Scientific) was added to each plate containing lymphocytes and incubated for 5 min at room temperature. Chloroform (0.2 mL) was added and the tubes shaken for 15 sec and then incubated at room temperature for 3 min. The mixture was then centrifuged at 12,000*g* for 15 min at 4°C. The supernatant was then transferred to a 1.5 mL tube and isopropyl alcohol (0.5 mL) was added to the tube. The mixture was incubated at room temperature for 10 min and centrifuged at 12,000*g* for 15 min at 4°C. The supernatant was then discarded, the pellet washed with 75% ethanol and then centrifuged at 7,500*g* for 5 min at 4°C. The supernatant was again discarded, the pellet partially dried, and then dissolved in RNase‐free water, and stored at ‐70°C.

### Human lymphocyte protein isolation

Protein was isolated as previously described (Toby et al. 2010; Jin et al. 2015). Briefly, cells were centrifuged and then washed with phosphate‐buffered saline (PBS) twice, and ice‐cold lysis buffer (20 mmol/L HEPES, pH 7.4, 50 mmol/L *β*‐glycerophosphate, 2 mmol/L EGTA, 1 mmol/L DTT, 10 mmol/L NaF, 1 mmol/L Na_3_VO_4_, 1% Triton X‐100, 10% glycerol) was added. Thirty minutes prior to use, the following protease inhibitors were added to each mL of lysis buffer: 2 *μ*g aprotinin, 5 *μ*g leupeptin, 0.7 *μ*g pepstatin A, and 174 *μ*g phenylmethylsulfonyl fluoride. The lysates were placed on ice for 30 min and then were centrifuged at 12,000*g* for 10 min at 4°C. The supernatant was transferred to new Eppendorf tubes and stored at −70°C for subsequent western blot analysis. Total protein concentration was determined by the Bradford method using a commercial assay kit (Bio‐Rad, Hercules, CA).

### RT‐PCR

RT‐PCR was performed as previously described (Nelin et al. 2002; Chen et al. 2009; Jin et al. 2015). Total RNA (2 *μ*g) was treated with DNase first and then reverse transcribed with 0.5 *μ*g random primer, 8 units dNTP, in 1x buffer (Promega, Madison, WI), and RNase‐free water, for a total volume of 20 *μ*L. Samples were incubated in a Thermal Cycler (Bio‐Rad) according to the manufacturer's procedure and stored at −20°C. Multiplex PCR for the expression of *ARG1*,* ARG2*, and *iNOS* was internally standardized by direct comparison to 18S ribosomal RNA expression in the same reactions. PCR reactions (total volume of 20 *μ*L) contained 1 *μ*L of RT product, PowerUp SYBR Green master mix, and 0.5 *μ*mol/L forward and reverse primers for each gene (Thermo Fisher). *ARG1* was amplified using the forward primer: 5′ TTGGCAATTG GAAGCATCTCTGGC 3′ and the reverse primer: 5′ TCCACTTGTGGTTGTCAGTGGAGT 3′. *ARG2* was amplified using the forward primer: 5′ TTAGCAGAGCTGTGTCAGATGGCT 3′ and the reverse primer: 5′ GGGCATCAACCCAGACAACACAAA 3′. *iNOS* was amplified using the forward primer: 5′ GCGTTACTCCACCAACAATGGCAA 3′ and the reverse primer: 5′ ATAGCGGATGAGCTGAGCATTCCA 3′. Negative controls containing reaction mixture and primers without cDNA were performed for each reaction to confirm that primers and reaction mixtures were without template contamination. The real‐time PCR reaction was performed according to the manufacturer's instruction using Mastercycler RealPlex 4 (Eppendorf, Hauppauge, NY). 18S was used as the control gene and was amplified using the forward primer (5′ CCAGAGCGAAA GCATTTGCCAAGA 3′) and the reverse primer (5′ TCGGCATCGTTTATGGTCGGAACT 3′). The relative mRNA expression levels were calculated using ΔΔC_*t*_ method (Livak and Schmittgen 2001).

### Western blot analysis

Cell lysates were assayed for arginase I, arginase II, cleaved caspase‐3, cleaved caspase‐8, or cleaved caspase‐9 proteins by western blot analysis as previously described (Nelin et al. 2007; Toby et al. 2010; Chen et al. 2012; Jin et al. 2015). Briefly, aliquots of lymphocyte lysates containing equal amounts of protein were diluted with SDS sample buffer and reducing agent, heated to 95°C for 5 min, and then centrifuged at 10,000*g* at room temperature for 2 min. Aliquots of the supernatant were used for SDS‐PAGE. The proteins were transferred to polyvinylidene difluoride membranes and blocked for 1 h in PBS with 0.1% Tween (PBS‐T) containing 5% nonfat dried milk. The membranes were then incubated with the primary antibody: arginase I or arginase II (1:500) (Santa Cruz Biotechnology Inc., Dallas, TX), cleaved caspase‐3, cleaved caspase‐8, or cleaved caspase‐9 antibody (1:1000) (Cell Signaling Inc., Danvers, MA) overnight at 4°C. The membranes were subsequently washed three times with PBS‐T, and then incubated with horseradish peroxidase (HRP)‐conjugated goat anti‐rabbit IgG (Bio‐Rad) or HRP‐conjugated goat anti‐mouse IgG (Bio‐Rad) for 1 h. After washing the membranes three times with PBS‐T, the protein bands were visualized using enhanced chemiluminescence (Luminata Classico or Forte Western HRP substrate, Millipore Corporation, Billerica, CA) and quantified using densitometry (VisionWorksLS Analysis Software; UVP LLC, Upland, CA). To control for protein loading, blots were stripped using a Western Re‐Probe buffer (G‐Biosciences, St. Louis, MO), and the blots were re‐probed for *β*‐actin (1:10,000) (Sigma‐Aldrich, St. Louis, MO), total caspase‐8, or total caspase‐9 (1:1,000) (Cell Signaling Inc) as described above.

### Inhibition of iNOS with l‐NAME

Lymphocytes (TT or GG) were stimulated with IL‐4, IL‐13, and PMA, treated with either vehicle or 300 *μ*mol/L l‐NAME (Sigma‐Aldrich), and added to the media for 24 h. Cell protein was harvested for determination of densitometry levels of cleaved caspase‐3, cleaved caspase‐8 and total caspase‐8, or cleaved caspase‐9 and total caspase‐9.

### Nitrite assay

Cell media was assayed for concentrations of nitrite (NO_2_^−^) using a chemiluminescence NO analyzer (Sievers, Boulder, CO), as previously described (Nelin et al. 2007; Jin et al. 2015) Briefly, 100 *μ*L of sample was placed in a reaction chamber containing a mixture of NaI in glacial acetic acid to reduce NO_2_^−^ to NO. The NO gas was carried into the NO analyzer using a constant flow of He gas. The analyzer was calibrated using a NaNO_2_ standard curve. Nitrite was measured in these experiments because the cell culture media contains relatively large quantities of calcium nitrate, therefore nitrite measurement is more sensitive for changes in NO production (Chicoine et al. 2004).

### Urea assay

Cell media was assayed in duplicate for urea concentration colorimetrically, as described previously (Toby et al. 2010; Jin et al. 2015). Briefly, 100 *μ*L sample was added to 3 mL chromogenic reagent (5 mg of thiosemicarbazide, 250 mg of diacetyl monoxime, 37.5 mg FeCl_3_ in 150 mL 25% (v/v) H_2_SO_4_, 20% (v/v) H_3_PO_4_). After 1 h at 37°C, the mixtures were vortexed and then boiled at 100°C for 5 min. The mixtures were cooled to room temperature and the absorbance (530 nm) was determined and compared against a urea standard curve.

### Proliferation assay

Lymphocytes (wild type) were seeded in 6‐well plates (1.6 × 10^5^ cells/well) in RPMI 1640 (with 10% FBS and 1% penicillin/streptomycin) with IL‐4, IL‐13, and PMA and incubated in 21% O_2_‐5% CO_2_‐balance N_2_ for 1, 2, 3, and 4 days. At the end of the experiment, the adherent cells were trypsinized and viable cells were counted using trypan blue exclusion, as described previously (Toby et al. 2010). In a second set of studies, 1.6 × 10^5^ cells/well of either TT or GG lymphocytes were seeded in 6‐well plates and incubated for 48 h. Viable cell numbers were counted using trypan blue exclusion.

### Statistical analysis

Values are shown as mean ± SE. An unpaired *t*‐test was used to compare data between genotypes (SigmaStat, Jandel Scientific, Carlsbad, CA). Linear regression was used to determine the relationship between nitrites and cleaved caspase‐3 (SigmaStat, Jandel Scientific). Differences were considered significant at *P* < 0.05.

## Results

### Arginase expression was not different between genotypes

**Figure 1:**
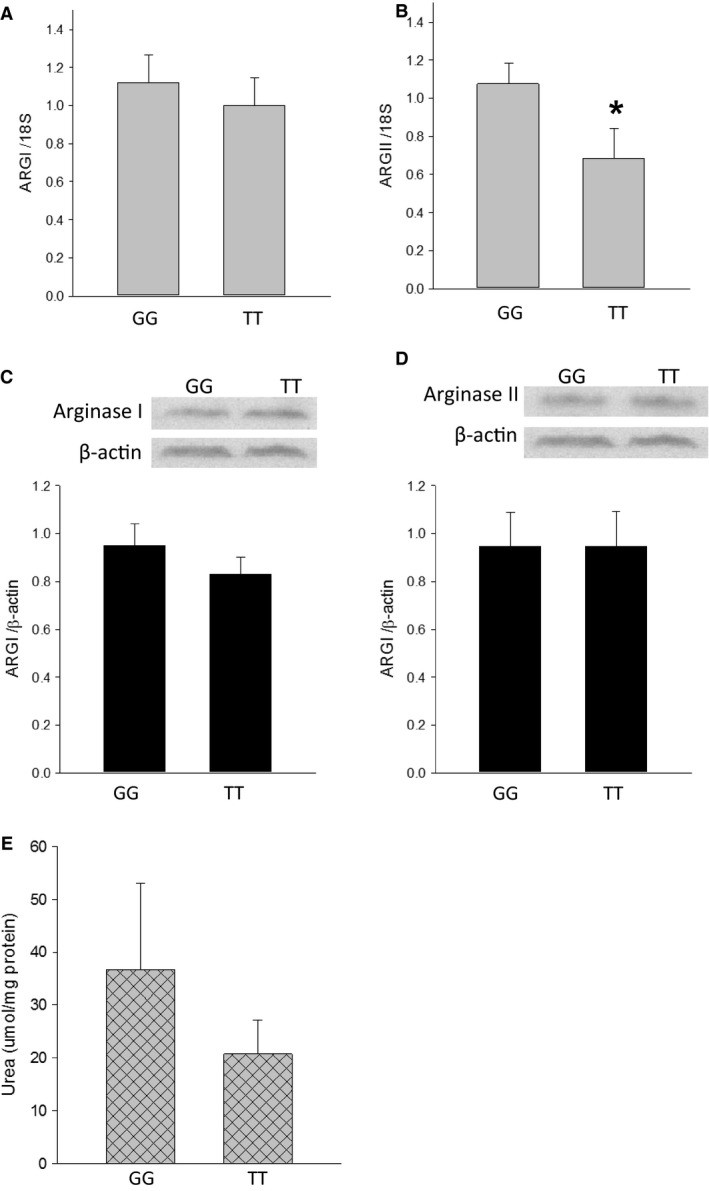
There was no difference in arginase I expression between genotypes. (A) Arginase I mRNA were not different in human lymphocytes with the *ARG1* rs2781666 single‐nucleotide polymorphism (SNP) (TT) (*N* = 9) as compared to wild type (GG) (*N* = 14). (B) Arginase II mRNA was lower in human lymphocytes with the *ARG1* rs2781666 SNP (TT) (*N* = 9) as compared to wild type (GG) (*N* = 14) (*P < 0.05). (C) Representative western blots and densitometries for arginase I normalized to *β*‐actin. Arginase I protein levels were not different in human lymphocytes with the *ARG1* rs2781666 SNP (TT) (*n* = 9) as compared to wild type (GG) (*N* = 14) allele. (D) Representative western blots and densitometries for arginase II normalized to Beta actin. Arginase II protein levels were not different in human lymphocytes with the *ARG1* rs2781666 SNP (TT) (*N* = 9) as compared to wild type (GG) (*N* = 14). (E) Urea levels tended to be lower in the media from lymphocytes with the TT genotype (*N* = 7) than in those from lymphocytes with the GG genotype (*N* = 8).

Lymphocytes isolated from patient cord blood genotyped as either SNP (TT) or wild type (GG) were stimulated with IL‐4, IL‐13, and PMA for 24 h. Then the cells were harvested either for mRNA or protein. *ARG1* and *ARG2* mRNA were determined by qPCR, and no difference was found in *ARG1* mRNA between the GG and the TT genotypes (Fig. 1A). *ARG2* mRNA was lower in the lymphocytes with the TT genotype than in those with the GG genotype (Fig. 1B). However, there were no differences between genotypes in the levels of arginase I or arginase II protein as determined by western blot (Fig. 1C and D). Although, there was a great deal of variability in the urea measurements, there was a trend toward lower urea production by the TT lymphocytes than by the GG lymphocytes (Fig. 1E).

### Nitric oxide production was greater in TT lymphocytes despite similar levels of iNOS expression

**Figure 2:**
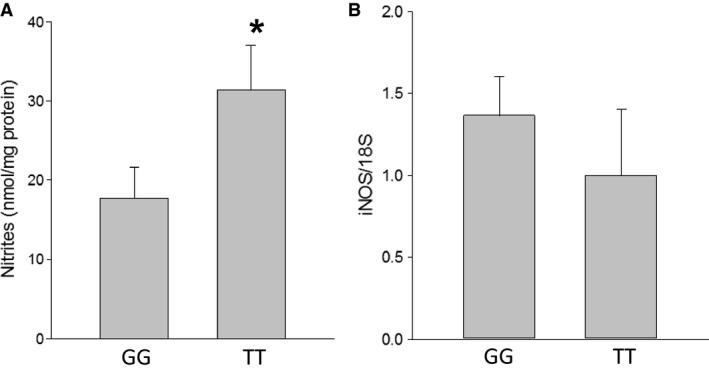
Nitrites were greater in the media from stimulated human lymphocytes with *ARG1* rs2781666 single nucleotide polymorphism (SNP) (TT) than in wild type (GG) lymphocytes. (A) Nitrites were measured in the media by chemiluminescence and then normalized to protein concentration (in mg). Nitrites were greater in lymphocytes with the *ARG1* rs2781666 SNP (TT) (*N* = 9) as compared to wild type (GG) (*N* = 15) (*P < 0.05). (B) The difference in NO production was not due to differences in iNOS expression. iNOS mRNA levels were not different in human lymphocytes with the *ARG1* rs2781666 SNP (TT) (*N* = 9) as compared to wild type (GG) (*N* = 15).

Stimulated lymphocytes from patients with the TT genotype had greater NO production (*P* < 0.05) than did stimulated lymphocytes from patients with the GG genotype (Fig. 2A). To determine whether the greater NO production in patient lymphocytes with the TT genotype was due to greater iNOS levels, we examined iNOS mRNA levels from stimulated lymphocytes from patients homozygous for TT or GG using qPCR. We found that there was no difference in iNOS mRNA levels between the two genotypes (Fig. 2B), demonstrating that the difference in NO production between genotypes was not due to greater iNOS expression in the TT lymphocytes.

### Proliferation was lower in stimulated human lymphocytes with the TT genotype

**Figure 3:**
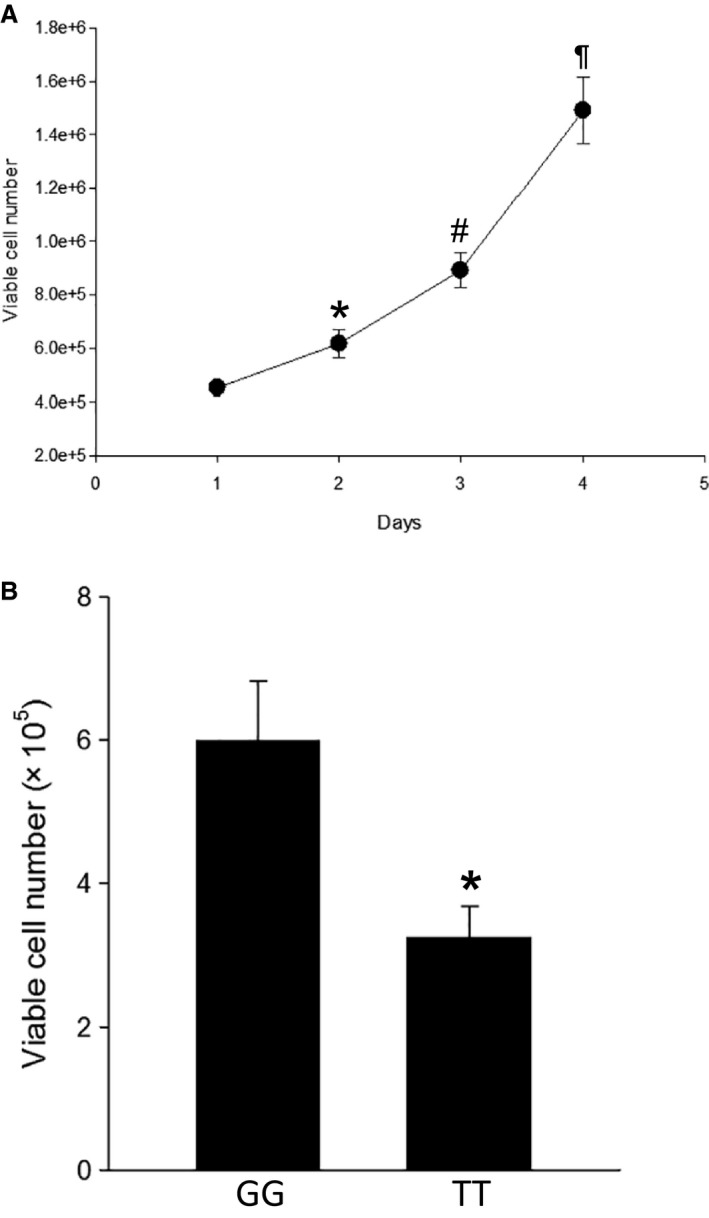
Viable cell numbers were lower after 48 h in culture for lymphocytes with the *ARG1* rs2781666 single‐nucleotide polymorphism (SNP) (TT) than in wild type (GG) lymphocytes. (A) Viable cell numbers for wild type (GG)‐stimulated lymphocytes were determined by trypan blue exclusion after 1, 2, 3, or 4 days in culture after seeding 1.6 × 10^5^ in each well of 6‐well plates. Viable cell number for lymphocytes increased for each day of growth; from day 1 to 2 (*P < 0.05), from day 2 to 3 (#*P* < 0.05), and from day 3 to 4 (¶*P* < 0.05). (B) Lymphocytes with the *ARG1* rs2781666 SNP (TT) (*N* = 9) had fewer viable cells than did wild type (GG) (*N* = 15) lymphocytes after 48 h in culture (*P < 0.05). Viable cell number was determined by trypan blue exclusion 48 h after seeding 1 × 10^5^ cells in each well of 6‐well plates.

First, we determined the rate of proliferation in stimulated wild type (GG) lymphocytes by measuring viable cell numbers using trypan blue exclusion assays, at day 1, 2, 3, or 4 after seeding 1.6 × 10^5^ cells in each well of 6‐well plates. We found that there was a significant increase in viable cell numbers each day from day 0 to day 4, from 1.6 × 10^5^ cells/well to a mean of 1.5 × 10^6^ cells/well on day 4 (Fig. 3A). This finding demonstrates active proliferation of these cultured lymphocytes. On the basis of these results, we next determined if there were differences in proliferation between the two genotypes. We used a 48 h time point since there was a fourfold increase in cell numbers from initial plating to 48 h. We found that, although the total number of viable lymphocytes increased from the plated number of 1.6 × 10^5^ in both genotypes after a 48 h incubation period, the number of viable cells was significantly lower after 48 h in culture for lymphocytes from the patients with the TT genotype than for lymphocytes from the patients with the GG genotype (Fig. 3B). These findings indicate that there was significantly less proliferation in cells with the TT genotype.

### Cleaved caspase‐3 protein levels were greater in stimulated human lymphocytes with the *ARG1* SNP (TT) than with the wild type (GG)

**Figure 4:**
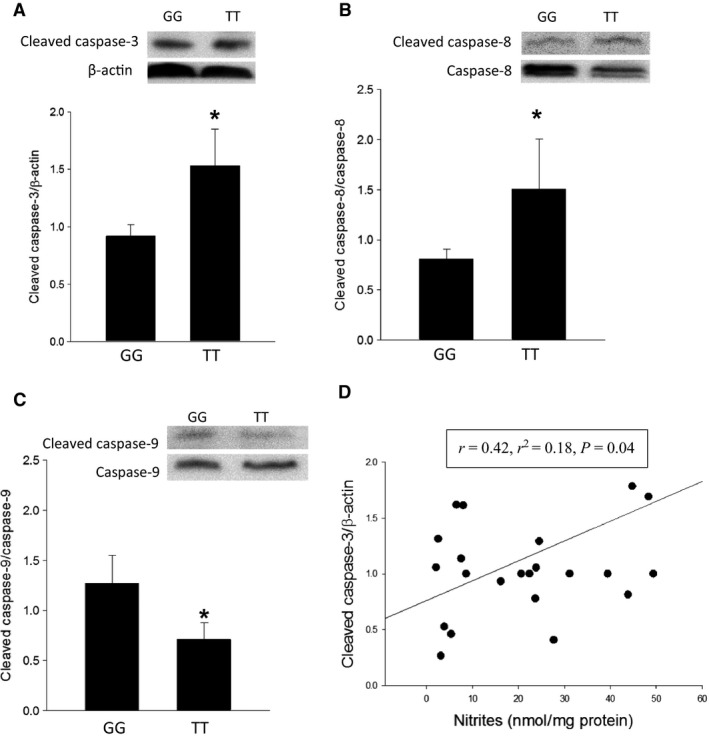
Cleaved caspase‐3 and cleaved caspase‐8 protein levels were greater in stimulated human lymphocytes with *ARG1* rs2781666 SNP (TT) as compared to similarly stimulated wild type (GG) lymphocytes. (A) Representative western blots for cleaved caspase‐3 and *β*‐actin, the bar graph shows densitometries for cleaved caspase‐3 normalized to *β*‐actin. Cleaved caspase‐3 protein levels were greater in human lymphocytes with *ARG1* rs2781666 SNP (TT) (*N* = 9) than in wild type (GG) lymphocytes (*N* = 14) (*P < 0.05). (B) Representative western blots for cleaved caspase‐8 and total caspase‐8, the bar graph shows densitometries for cleaved caspase‐8 normalized to total caspase‐8 levels. Cleaved caspase‐8 protein levels were greater in human lymphocytes with *ARG1* rs2781666 SNP (TT) (*N* = 9) than in the wild type (GG) lymphocytes (*N* = 16) (*P < 0.05). (C) Representative western blots for cleaved caspase‐9 and total caspase‐9, the bar graph shows densitometries for cleaved caspase‐9 normalized to caspase‐9 levels. Cleaved caspase‐9 protein levels were lower in human lymphocytes with the *ARG1* rs2781666 SNP (TT) (*N* = 9) than in wild type (GG) lymphocytes (*N* = 16) (*P < 0.05). (D) Media concentration of nitrites plotted against cleaved caspase‐3 densitometry normalized to *β*‐actin. Linear regression demonstrated a positive correlation between nitrite concentration and cleaved caspase‐3 levels (*r* = 0.42, *r* ^2^ = 0.18, *P* = 0.04).

Since the increase in viable cell numbers depends on the balance between apoptosis and proliferation, and given that the TT lymphocytes had fewer viable cells and greater NO production than did the GG lymphocytes, we examined apoptosis in these lymphocytes. Lymphocytes with either the TT genotype or the GG genotype were stimulated with IL‐4, IL‐13, and PMA for 24 h and protein was harvested for western blotting and probed for cleaved caspase‐3 (effector caspase), cleaved caspase‐8 (extrinsic receptor apoptosis pathway), or cleaved caspase‐9 (intrinsic mitochondrial apoptosis pathway). Stimulated TT lymphocytes had significantly greater levels of cleaved caspase‐3 protein than did stimulated GG lymphocytes (Fig. 4A). Stimulated TT lymphocytes also had significantly greater levels of cleaved caspase‐8 protein than did GG lymphocytes (Fig. 4B). We also found that TT lymphocytes had lower levels of cleaved caspase‐9 protein than did GG lymphocytes (Fig. 4C). To determine if the levels of cleaved caspase‐3 were directly correlated with NO production, we plotted cleaved caspase‐3 levels as a function of media nitrite concentration. We did find a positive, although relatively weak, correlation (*r* = 0.41, *r* ^2^ = 0.18, *P* = 0.04) between nitrite levels in the media and cleaved caspase‐3 protein levels in stimulated human lymphocytes (Fig. 4D).

### Inhibition of NOS attenuated cleaved caspase‐3 protein levels in TT lymphocytes

**Figure 5:**
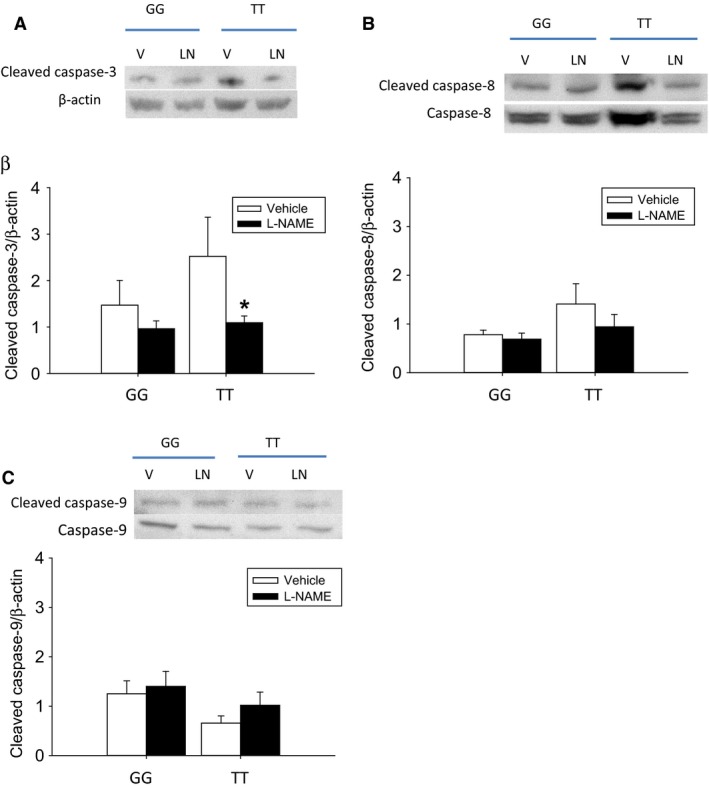
l‐NAME attenuates cleaved caspase‐3 protein levels in stimulated human lymphocytes with *ARG1* rs2781666 SNP (TT). (A) Representative western blots for cleaved caspase‐3 and *β*‐actin, the bar graph shows the densitometries for cleaved caspase‐3 normalized to *β*‐actin. Cleaved caspase‐3 protein levels in lymphocytes with *ARG1* rs2781666 SNP (TT) were lower in lymphocytes treated with l‐NAME (*N* = 9) than in vehicle‐treated lymphocytes (*N* = 9) (*P < 0.05). There were no differences between the vehicle and l‐NAME‐treated GG lymphocytes. (B) Representative western blots for cleaved caspase‐8 and total caspase‐8, the bar graph shows the densitometries for cleaved caspase‐8 normalized to total caspase‐8. Treatment with l‐NAME did not significantly change levels of cleaved caspase‐8 protein (*N* = 9 in each treatment group). (C) Representative western blots for cleaved caspase‐9 and total caspase‐9, the bar graph shows the densitometries for cleaved caspase‐9 normalized to total caspase‐9. Treatment with l‐NAME did not significantly change the levels of cleaved caspase‐9 protein (*N* = 9 in each treatment group). V, vehicle; LN, l‐NAME.

To further examine the role of NO production in the greater apoptosis seen in the TT lymphocytes, we used the NOS inhibitor, l‐NAME (300 *μ*mol/L l‐NAME or vehicle was added to the media). After 24 h, protein was harvested for determination of cleaved caspase‐3, cleaved caspase‐8, or cleaved caspase‐9 levels. The stimulated TT lymphocytes treated with l‐NAME had significantly lower levels of cleaved caspase‐3 protein than did the stimulated TT lymphocytes treated with vehicle (Fig. 5A). There was no difference in cleaved caspase‐3 levels in the GG lymphocytes treated with l‐NAME compared to vehicle‐treated stimulated GG lymphocytes (Fig. 5A). Similarly, levels of cleaved caspase‐3 protein were not different between l‐NAME‐treated stimulated GG lymphocytes and l‐NAME‐treated stimulated TT lymphocytes (Fig. 5A). Although, there was a trend toward lower cleaved caspase‐8 protein levels in the TT lymphocytes with l‐NAME treatment, this did not reach statistical significance (Fig. 5B). We found no significant effect of l‐NAME on cleaved caspase‐9 protein levels in stimulated lymphocytes with either genotype (Fig. 5C).

## Discussion

The main findings of this study were that lymphocytes from patients homozygous for the *ARG1* rs2781666 SNP (TT) compared to lymphocytes from patients with the GG genotype had: (1) similar expression levels of arginase I, arginase II, although there was a tendency toward lower urea production; (2) greater NO production although the expression levels of iNOS were similar; (3) less cellular proliferation; and (4) greater levels of cleaved caspase‐3 and the levels of cleaved caspase‐3 were significantly attenuated by NOS inhibition.

Lymphocytes from patients with the *ARG1* rs2781666 SNP (TT) had greater NO production than did wild type (GG) lymphocytes, without an increase in iNOS expression. Furthermore, there were no differences in arginase protein expression between genotypes; but, there was a tendency toward lower urea production in the TT lymphocytes. These findings are consistent with the notion that greater l‐arginine bioavailability to iNOS is involved in the greater NO production observed in the TT lymphocytes (Chicoine et al. 2004; Jin et al. 2015). l‐arginine is the substrate for both NOS and arginase. The role of arginase in limiting l‐arginine bioavailability to NOS has been described in several cell types (Hey et al. 1997; Chicoine et al. 2004; Li et al. 2001). We have also shown that iNOS overexpression in pulmonary endothelial cells decreased arginase activity (Stanley et al. 2006). Therefore, taken together, these data are consistent with lower arginase activity in the presence of the *ARG1* SNP allowing for greater l‐arginine bioavailability to NOS leading to greater NO production, and greater NO production could potentially be involved in preventing vascular remodeling and/or reversing vasoconstriction that leads to protection from the development of PH in BPD.

The only cell type that we had access to from patients with the SNP of interest were lymphocytes. We found that these umbilical cord‐derived lymphocytes proliferated over time in culture. Importantly, we found that the lymphocytes from patients with the *ARG1* SNP (TT) had reduced viable cell numbers in culture compared to cultured lymphocytes from patients with the GG genotype. We have previously shown in human pulmonary microvascular endothelial cells and smooth muscle cells that cellular proliferation is dependent on arginase activity (Chen et al. 2009, 2012; Toby et al. 2010). When we measured urea production, as a marker of arginase activity in stimulated lymphocytes, we found a trend toward lower urea production in the TT lymphocytes than in the GG lymphocytes, indicating lower arginase activity. Although there was a great deal of variability in the urea production, particularly in the lymphocytes from patients with the GG genotype, this may have resulted from variability in the response to the PMA, IL‐4, and IL‐13 stimulation. Taken together, these data are consistent with the concept that the *ARG1* SNP (TT) resulted in decreased l‐arginine utilization by arginase, which would lead to decreased l‐ornithine production, decreased polyamine and proline production, and fewer viable cells in culture.

Although proliferation is an important part of vascular remodeling, the process also depends on the balance between proliferation and apoptosis. We found that lymphocytes from patients with the TT genotype had greater levels of the activated effector caspase, cleaved caspase‐3, than did lymphocytes from patients with the GG genotype. Furthermore, we found that the greater cleaved caspase‐3 protein expression in the TT lymphocytes was prevented by inhibition of NOS. This finding is consistent with the notion that the increased NO production in TT lymphocytes results in greater apoptosis and is also consistent with several studies in many other cell types showing that NO is pro‐apoptotic (Fukuo et al. 1996; Geng et al. 1996; Pollman et al. 1996; Iwashina et al. 1998). In pulmonary endothelial cells, we have shown that overexpression of iNOS led to significantly greater levels of NO production and significantly lower numbers of viable cells in culture (Stanley et al. 2006). Thus, it is likely that a major contributor to the lower viable cell numbers in the TT lymphocytes was greater NO‐mediated apoptosis. Therefore, we postulate that the *ARG1* SNP (TT) may be protective against the development of PH in BPD patients by promoting NO‐mediated apoptosis. However, the role of the *ARG1* SNP in apoptosis of cells in the pulmonary vascular wall on the development of BPD‐associated PH is an important area of further research.

A limitation of this study is the use of lymphocytes, instead of a cell type from the vascular wall. However, neonatal patients are difficult to study because procedures, such as bronchoscopy, lung biopsy, catheterization, etc., are very difficult to perform given the small size of these patients. Therefore, the only cell type that we had access to from these patients are the lymphocytes isolated from cord blood specimens. We studied lymphocytes (GG and TT) from neonatal patients, and did not require genetic manipulation of the cells following isolation. There is no evidence that these cells are equivalent in terms of responses to various stimuli, but lymphocytes do express arginase I and II, iNOS, and cleaved caspase 3, 8, and 9, as do endothelial cells and vascular smooth muscle cells. Although, not definitive, our study provides proof of concept and demonstrates the need for further studies on the role of arginase mutations in the development of PH in BPD.

In conclusion, our findings support our hypothesis that BPD patients with the *ARG1* rs2781666 SNP are protected against PH at least in part by greater NO production via greater l‐arginine bioavailability to NOS. We postulate that the greater l‐arginine bioavailability to NOS is through decreased activity of arginase I. Shifting the balance toward apoptosis and away from proliferation in patients with the *ARG1* rs2781666 SNP may result in the attenuation and/or amelioration of vascular remodeling. Furthermore, our data suggest that arginase I inhibition potentially represents a novel therapeutic target for the prevention and/or treatment of BPD‐associated PH.

## Conflicts of Interest

No conflicts of interest are declared by the authors.
